# Synergies between Intrinsic and Synaptic Plasticity Based on Information Theoretic Learning

**DOI:** 10.1371/journal.pone.0062894

**Published:** 2013-05-09

**Authors:** Yuke Li, Chunguang Li

**Affiliations:** Department of Information Science and Electronic Engineering, Zhejiang University, Hangzhou, People's Republic of China; University of Sheffield, United Kingdom

## Abstract

In experimental and theoretical neuroscience, synaptic plasticity has dominated the area of neural plasticity for a very long time. Recently, neuronal intrinsic plasticity (IP) has become a hot topic in this area. IP is sometimes thought to be an information-maximization mechanism. However, it is still unclear how IP affects the performance of artificial neural networks in supervised learning applications. From an information-theoretical perspective, the error-entropy minimization (MEE) algorithm has newly been proposed as an efficient training method. In this study, we propose a synergistic learning algorithm combining the MEE algorithm as the synaptic plasticity rule and an information-maximization algorithm as the intrinsic plasticity rule. We consider both feedforward and recurrent neural networks and study the interactions between intrinsic and synaptic plasticity. Simulations indicate that the intrinsic plasticity rule can improve the performance of artificial neural networks trained by the MEE algorithm.

## Introduction

Artificial neural networks with nonlinear processing elements are designed to deal with the troublesome problem of nonlinear and nonstationary signal processing. In a supervised learning problem, we are provided with a training data set containing the input, 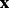, and the desired output (target), 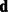, and we aim at finding the input-output mapping that models the complicated relationship between 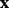 and 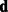. To solve such a problem, we can employ an artificial neural network trained by an appropriate learning algorithm to infer the mapping implied by the training data. Most current learning algorithms for artificial neural networks in applications rely on updating the connection weights 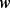 among neurons. This is often done with the aim of minimizing the mean square error (MSE) between the network output 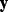 and the desired output 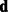 over all input-target pairs, where the error is defined as 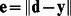. However, the MSE criterion takes into account only the first two moments of the error distribution, making it ill-suited to non-linear applications in which the errors are not normally distributed. The error entropy criterion (EEC) has been proposed on information-theoretic grounds by Principe et al. as an alternative cost function that takes into account the full distribution of errors [1]. This is the form of synaptic plasticity we consider in this article.

So far, experimental and theoretical studies on neural plasticity have mostly focused on synaptic plasticity, which is in accordance with Hebb's idea that memories are stored in the synaptic weights and learning is the process that changes those synaptic weights. Interestingly, recent experimental results have revealed that neurons are also capable of changing their intrinsic excitability to match the dramatic change of the level of received synaptic input [2]–[9]. This novel neural mechanism is referred to as *intrinsic plasticity* (IP). IP is hypothesized to maximize the information capacity while maintaining an individual neuron's homeostasis of its mean firing rate level [10]–[12]. To better understand the role IP might play in learning and memory, several IP rules [10], [11], [13], [14] were proposed that bring the firing rate distribution into a desired one with a relatively low activity level as observed in visual cortical neurons [15]. Actually, upon neglecting the energy constraint, these IP rules [10], [11], [13], [14] are closely related to the single-neuron case of Bell and Sejnowski's information-maximization algorithm [16]. When the input-output mutual information is maximized by this learning algorithm, a neuron uses all of its possible response levels equally and uses the steep parts of the activation function to respond to the high density parts of the input probability density function (PDF); therefore, this information-maximization algorithm enhances the discriminative ability of the neuron.

The two plasticity mechanisms, intrinsic plasticity and synaptic plasticity, have been studied mostly separately. We are wondering how these two plasticity mechanisms would cooperate in artificial neural networks to learn complex mappings. To this end, we combine Bell and Sejnowski's information-maximization algorithm [16] for intrinsic plasticity and the error-entropy minimization (MEE) algorithm [17] for synaptic plasticity, which we refer to as *synergistic information-theoretic learning*. We use the resulting synergistic procedure for training feedforward neural networks (FNN) and recurrent neural networks (RNN) and test them on two benchmark applications. For simplicity and clarity, we focus on the prediction problem in the presentation, but the learning algorithm can also be used for solving problems of regression, classification and so on. Simulations indicate that Bell and Sejnowski's algorithm is appropriate for the proposed synergistic learning scheme and that the MEE algorithm combined with IP outperforms the MEE algorithm considered in isolation.

## Materials and Methods

### Information-maximization Algorithm as an Intrinsic Plasticity Rule

Studying the effects of intrinsic plasticity on various neural functions and dynamics relies on modelling intrinsic plasticity. In [10], [11], [13], [14], several intrinsic plasticity rules were proposed, which bring the firing rate distribution into a desired one with a relatively low activity level as observed in visual cortical neurons [15]. In all these IP rules, the energy consumption of a biological neuron is considered as an important constraint. Keeping a low average output is critical for biological organisms due to energy expenditure, but it seems unnecessary for artificial neural networks such as the FNN and the RNN. In terms of choosing the IP rule in this situation, we prefer to emphasize the character of maximizing the information capacity. Neglecting the energy constraint in this study, we apply the information-maximization learning algorithm proposed by Bell and Sejnowski [16] as the intrinsic plasticity rule for individual neurons of artificial neural networks. This learning algorithm adjusts the slope and the bias of the activation function to maximize the mutual information between the input and the output of each neuron. As a result of this learning procedure, the activation function is adapted to match the input distribution, i.e., sensitive parts of the activation function respond to high density parts of the input probability density function. The sensitivity is characterized by the slope of the response curve and steep parts are more sensitive than flat parts. For the tanh activation function of the 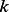
th neuron 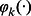,
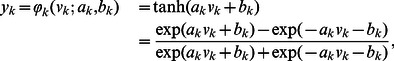
(1)where 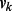 is the input of the 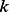
th neuron, 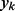 is the output of the 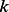
th neuron, 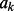 represents the sensitivity of the activation function and 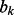 is the bias. The corresponding information-maximization learning algorithm can be obtained as follows
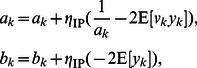
(2)where 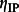 is the learning rate of intrinsic plasticity. For a training set including 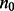 samples, the input-output pairs of the 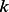
th neuron, 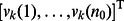 and 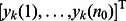, are used to estimate the expected values 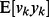 and 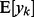. This batch version of the information-maximization rule can be derived directly from the objective of entropy maximization (“online” equivalent). Note that this learning rule neglects the recurrent interactions that may exist in the network, such that the entropy of the output of the 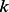
th unit is assumed to depend only on 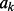 and 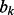, but not on any other 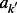 and 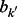 for 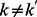.

We apply this information-maximization rule as the intrinsic plasticity rule for artificial neural networks in this paper. Note that the original information-maximization rule in [16] is an online weight update rule for ICA. For simplicity, in all of the following presentations and simulations, the tanh function is chosen as the activation function and the corresponding intrinsic plasticity rule is applied unless stated otherwise.

### MEE Algorithm as a Synaptic Plasticity Rule

As mentioned above, the MSE criterion considers only the first two moments of the error distribution, thus it is ill-suited to non-linear applications in which the errors are not normally distributed. Recently, the error entropy (EEC) criterion based on ideas from information theory has been proposed as an alternative cost function for learning [1]. EEC aims at removing as much uncertainty as possible from the error signal, and this can be accomplished by calculating the entropy of the error and minimizing it with respect to the connection weights. In the ideal case, all the uncertainty in the error is removed and the error probability density function is a delta function. This method is called Minimization of the Error Entropy (MEE) [17].

In applications, Renyi's quadratic entropy, 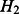, is often applied instead of Shannon's entropy [1]. We can easily use Renyi's quadratic entropy to derive a learning rule. For a probability density function 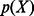 where 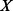 is a continuous random variable, the formula for Renyi's quadratic entropy is given by
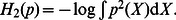
(3)One can then define a quadratic potential 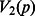, such that
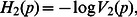
(4)where



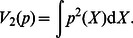
(5)Thus, the minimization of Renyi's quadratic entropy in Eq. (3) is equivalent to the maximization of the information potential in Eq. (5). Importantly, Eq. (5) may be interpreted as an expectation of the function 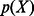 under itself, that is, 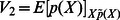. This means that, provided one can estimate 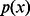 for any sample 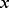, one may subsequently estimate 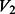 through simple Monte-Carlo averaging of 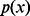 over many independent samples from 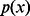 (i.e. the data set). Here, we use Gaussian kernel density estimation with bandwidth 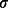, 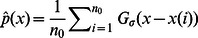, where 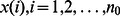 are 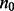 samples from the true underlying distribution 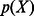, 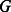 denotes the Gaussian kernel function, and 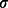 represents the kernel size for probability density function estimation [18]. Assuming Gaussian kernels and substituting this in the quadratic entropy expression Eq. (5), we get the following estimator for 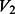 [1],
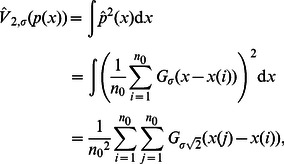
(6)where 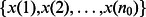 is a set of data samples. With the steepest ascent approach, the training algorithm for weight updating to maximize the quadratic information potential of the error 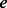, 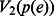, becomes
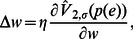
(7)where 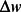 denotes the change of the weight 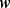 and 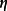 is the learning rate. The gradient of the quadratic information potential with respect to the connection weight is



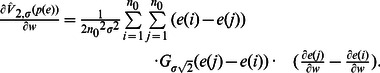
(8)In training with entropy-based criteria, one important point to note is that since entropy does not change with the mean of the distribution the algorithm will converge to a set of optimal weights that may not yield zero-mean error. This problem can be easily solved by adding a bias to the final output to yield zero mean error over training data set after the training procedure ends [19].

We now introduce our synergistic information-theoretic learning algorithm, which is the simple combination of the IP rule of Eq. (2) and the synaptic plasticity rule of Eq. (7).

### Synergistic Information-theoretic Learning

From the perspective of information maximization, there are potential advantages of intrinsic plasticity in training artificial neural networks. For traditional weight update learning algorithms (synaptic plasticity rules), the activation functions of neurons are fixed during the training procedure. However, an invariant nonlinear activation function might be unsuitable for the input distribution. In an extreme case, the output of the neuron may be constantly found at saturation, and therefore carry very little information about the input. For real-world applications, the desired output distribution of a single neuron is far from these distributions with very low information. As we stated in the previous section, the information-maximization algorithm (the intrinsic plasticity rule) can adjust the shape of the activation function to match the input distribution and consequently increase the mutual information between the input and the output. Therefore, we hypothesize that the intrinsic plasticity rule might be beneficial to learning in artificial neural networks.

The MEE algorithm requires several samples to accurately estimate the information potential. We therefore perform batch (epochwise) learning iterations, whereby the weights 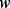 are updated according to Eq. (8), on the basis of the output-target pairs collected from all samples in the training set [20]. This allows for a correct estimation of the quadratic potential, and therefore more stable learning. Note that the exact form of the gradient in Eq. (8) depends on the network architecture, and is derived below for both feedforward and recurrent networks. Following this weight update, which we call the “synaptic stage” of a learning iteration, we update the parameters 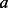 and 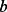 of the activation function 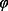 of each neuron to implement intrinsic plasticity according to Eq. (2). This we call the “intrinsic stage”. Note that the expected values 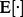 in Eq. (2) are again estimated from the input-output pairs collected from all samples in the training set. The synaptic and intrinsic stages together define one learning iteration (epoch), which we repeat until the stopping criterion is satisfied. In the following simulations, we stop the learning process after a certain number of learning iterations.

One may think that the effects of synaptic plasticity and intrinsic plasticity merely superpose in the learning process. In fact, we argue that they interact, which is why we call this combination “synergistic learning”. Indeed, the weight updating procedure affects the input of the neuron, and further influences the IP learning; the IP learning procedure affects the output of the neuron, and further influences the weight updating.

### Stability of Synergistic Learning

It has been noted that, in reservoir networks, due to the incremental nature of intrinsic plasticity (the value of parameter 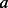 increases during learning), too large a value for 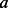 can cause unstable learning behavior (oscillations in the learning curve) and thus the performance might deteriorate as learning goes on [21]. This phenomenon is due to the cancellation effect, whereby high gains can be compensated by small synaptic weights. Nevertheless, with a proper IP learning rate, unstable behavior takes place only when the IP rule is applied for a very long training time; thus, IP learning can be kept stable before the stopping criterion of the cost function is satisfied [21].

### Construction of the FNN

**Figure 1:**
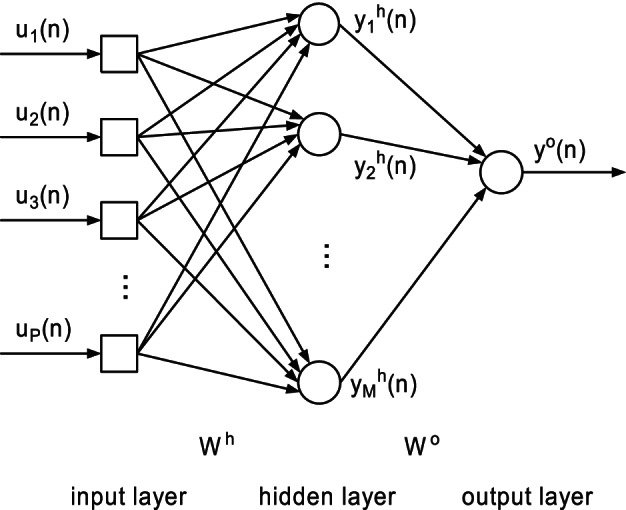
Structure of the feedforward neural networks.

In order to study the performance of the proposed synergistic learning algorithm, we first choose a general class of feedforward neural networks (FNN) as an example. As illustrated in Fig. 1, this neural network is composed of an input layer, a single hidden layer and an output layer. The activation function of each neuron is 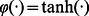. The network input consisting of 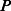 external elements can be described by the 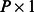 vector, 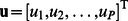. In the hidden layer, there are 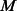 neurons (processing elements). Each neuron in the hidden layer receives the weighted sum of the network input 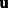. The output of these neurons, 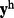, is described as 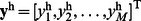, and is calculated by
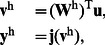
(9)where 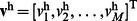, and 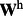 represents the 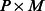 synaptic weight matrix connecting the input layer to the hidden one. An element 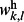 of this matrix represents the weight connection from the 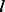
th input node to the 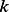
th hidden neuron. For the output layer, we consider only one neuron, which receives the weighted sum of the output of hidden neurons, 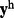, and produces the network output, 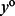. The calculation is described as
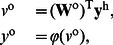
(10)where 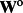 represents the 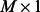 synaptic weight matrix linking the hidden layer to the output unit. An element 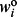 of this matrix represents the connection weight from the 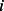
th hidden neuron to the final output.

### Synergistic Algorithm for the FNN

The difference between the desired output, 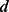, and the network output, 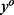, is defined as the error of the FNN, 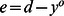. For the output layer of a single-output FNN, the derivative of the error 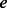 with respect to the weight 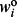 in the 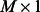 matrix 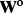 can be calculated as
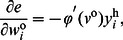
(11)where



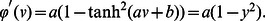
In a multi-layer FNN, a backpropagation algorithm is usually used to train the weight matrix from the input layer to the hidden layer, 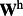. If the EEC cost function is used, the training algorithm is the MEE algorithm [22]. The derivative of the error 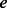 with respect to the weight 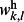 in the matrix 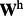 can be calculated as
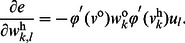
(12)By Eq. (11) and Eq. (12), the weight update rule in Eq. (7) and Eq. (8) can be calculated.

On the basis of the algorithm description in [23], the proposed synergistic learning algorithm for the FNN is summarized as follows:

#### Step 1. Initialization

Choose a random set of small values for the 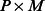 hidden layer weight matrix 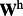 and the 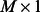 output layer weight matrix 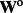. Set 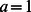 and 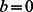 for each neuron. Let 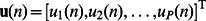 be the input signal and let 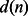 be the corresponding desired network output. The number of samples in the training set is 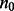, thus 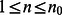.

#### Step 2. Repetition

The epochwise training procedure begins with 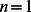. Repeat the following calculations with the input vector 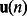 and the target output 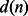 for 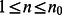


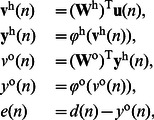
(13)where



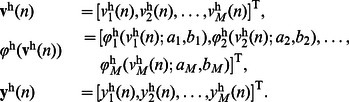



#### Step 3. Weight Matrix Update

Update the weight matrices 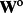 and 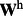 by the weight update algorithm. Calculation results of 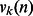 and 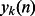 in Eq. (13) are used to compute the derivatives of the error with respect to the weight in Eq. (11) and Eq. (12); with the results of the derivatives and the errors 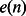 in Eq. (13), Eq. (8) can be computed and finally the weight matrices can be updated by Eq. (7).

#### Step 4. Activation Function Update

Update the parameters 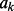 and 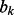 of the activation function 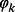 of the neuron 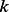 using the intrinsic plasticity rule described in Eq. (2) with all values of 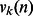 and 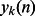. By the batch version of the IP rule, the parameters 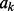 and 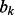 are only updated once during an epoch.

#### Step 5. Return or Stop

If the stopping criterion is satisfied, the training procedure is stopped; otherwise, set 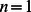 and return to Step 2.

### Construction of the RNN

**Figure 2:**
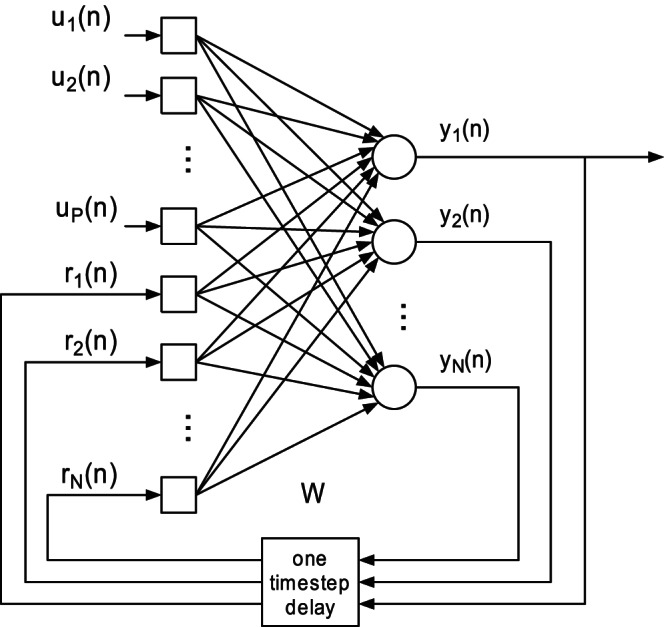
Structure of the recurrent neural networks.

In this section, we continue to study the proposed synergistic learning algorithm in a general class of recurrent neural networks [23], [24]. As illustrated in Fig. 2, the neural network contains 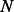 neurons. The input vector 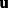 is comprised of the external signal vector of 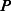 elements 
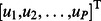
, and the feedback vector 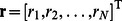. The feedback signal 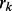 after a delay of one time unit is the output of the 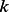
th neuron 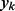, 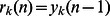, thus the feedback vector at the time point 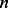 can be rewritten as 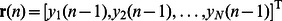. Then the input vector at the time point 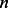 is given by
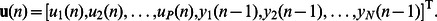



The 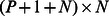 synaptic weight matrix of the recurrent network is represented by 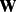. An element 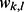 of this matrix represents the connection weight from the 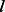
th input node to the 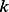
th neuron. With the input vector 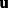 and the activation function 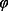, the 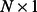 output vector 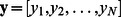 is calculated as
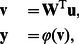
(14)where 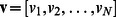 and 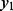 is the single output of the network.

### Synergistic Algorithm for the RNN

Following the approach of [25], a recursive learning algorithm can be derived for the recurrent neural network. Referring to [26], the gradients of the outputs of the neurons 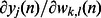 can be computed recursively as follows
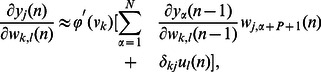
(15)where 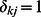 for 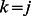, otherwise, 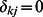. The initial state is 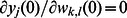. With the relationship 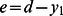, where 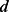 is the desired output and 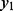 is the true output of the RNN, the partial derivative of the error with respect to the weight becomes



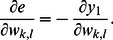
(16)By using Eq. (15) and Eq. (16), the weight update rule in Eq. (7) and Eq. (8) can be calculated.

The proposed synergistic learning algorithm for the RNN is summarized as follows:

#### Step 1. Initialization

Choose a random set of small values for the 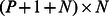 weight matrix 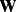 and the 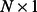 feedback vector 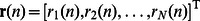. Set 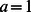 and 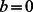 for all neurons. Obtain the 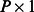 external input vector and the desired signal 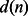 with 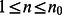.

#### Step 2. Repetition

The epochwise training procedure begins with 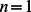. Input the external input vector, the feedback vector and the desired signal, and perform the following calculations
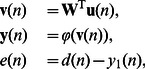
(17)where



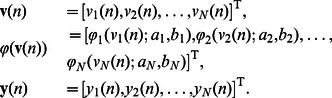



Let 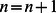, and set



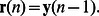
.

Repeat the calculation until 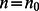.

#### Step 3. Weight Matrix Update

Update the weight matrix 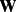 by the MEE learning algorithm. Calculation results of Eq. (17) of the current epoch are used to compute the derivatives of the error with respect to the weight in Eq. (15) and Eq. (16); with the results of the derivatives and the errors 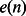 in Eq. (17), Eq. (8) can be computed and finally the weight matrix can be updated by Eq. (7).

#### Step 4. Activation Function Update

Update the parameters 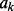 and 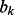 of the activation function 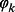 of the neuron 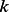 using the intrinsic plasticity rule described in Eq. (2). With the batch version of the IP rule, the parameters 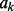 and 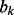 are only updated once during an epoch.

#### Step 5. Return or Stop

As one epoch ends, if the stopping criterion is satisfied, the training procedure is stopped; otherwise, set 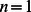 and 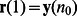, and return to Step 2.

## Results

The FNN and RNN are widely applicable to a set of problems such as regression and classification. As a typical example, we test the proposed synergistic learning algorithm on the single-step prediction of time series. For comparison, we also perform simulations for the MEE algorithm alone. The time series is denoted as 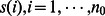. In the following simulations, two data sets of different time series are used. The first data set is the well-known Mackey-Glass chaotic time series, which often serves as a benchmark in testing prediction algorithms in the literature. The Mackey-Glass system (for 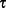 = 17) is described by the following differential equation
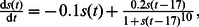
(18)which is a chaotic system modelling irregular behaviors in biological systems [27]. In our simulations, we use the Runge-Kutta method with time-step 0.1 to integrate Eq. (18), and then we draw samples at 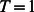
s interval to obtain the discrete time series. We use 300 samples for training and 10000 new samples generated from a different initial condition for testing. We use “MG” to denote this data set. The other data set is a speech signal obtained from an audio report in the program of “Scientific American 60 Seconds Science”. We name this data set “SS”. We use 300 samples from this speech signal for training and 10000 different samples for testing. The values of these two data sets are all normalized in the range [−1, 1].

### Results of the FNN

The 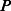 external time-delay signals serve as input of the FNN. At the 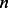
th time point, the input vector 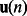 is described by the 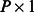 vector,



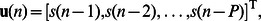
where 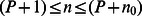. In the output layer, there is only one neuron. The actual prediction made by the feedforward neural network at time 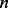 is the output of the FNN 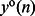, and the desired prediction is 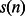.

In the following simulations, the elements of the initial weight matrices 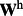 and 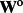 are randomly selected as small values uniformly distributed in [0, 0.05]. All numerical results in this section are averaged over 10 independent runs. The learning curves of these 10 runs are quite similar and the standard deviations of the learning results across the 10 independent runs are very small, and are therefore not shown here. As for many other learning algorithms, the convergence of the MEE-BP algorithm is slow when a small learning rate is adopted. Certainly we can increase the learning rate to accelerate the training process, but in this situation the learning curve tends to oscillate slightly at the latter stage of the training process. In this paper, we use a damping learning rate to make the learning process fast at the beginning and to prevent oscillations in the long run. The initial learning rate is set to 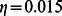 and the learning rate decreases exponentially from one epoch to the next, 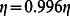. As we have mentioned in the previous section, a damping IP learning rate is also used to prevent unstable learning behavior during a long training process. The initial learning rate of IP in Eq. (2) is 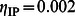, and the IP learning rate also decreases exponentially, 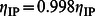.

**Figure 3:**
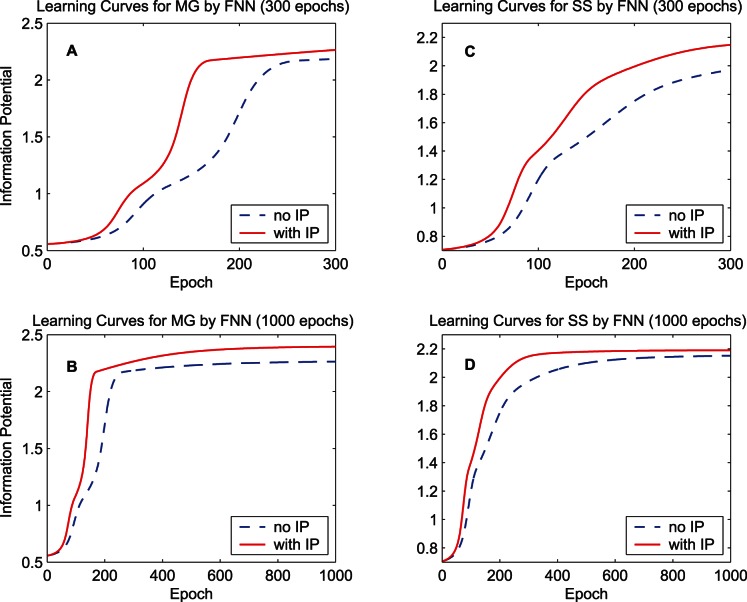
Learning curves of the quadratic information potential by the FNN. The dashed lines denote the learning curves of the MEE algorithm, and the solid lines denote the learning curves of the synergistic algorithm. (A) 300-epoch learning curves for the training data set “MG”. (B) 1000-epoch learning curves of “MG”. (C) 300-epoch learning curves for the training data set “SS”. (D) 1000-epoch learning curves of “SS”.

**Figure 4:**
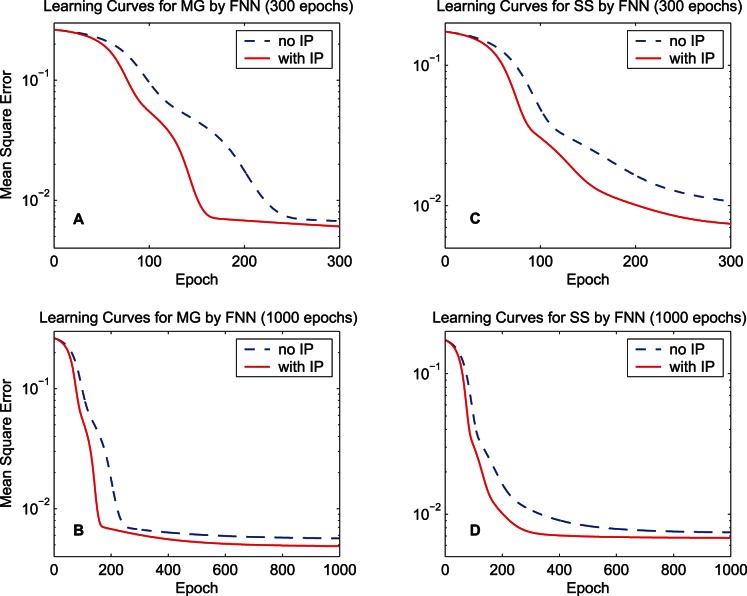
Learning curves of the mean square error by the FNN. The dashed lines denote the learning curves of the MEE algorithm, and the solid lines denote the learning curves of the synergistic algorithm. (A) 300-epoch learning curves for the training data set “MG”. (B) 1000-epoch learning curves of “MG”. (C) 300-epoch learning curves for the training data set “SS”. (D) 1000-epoch learning curves of “SS”.

The first simulation compares the learning curves of the MEE algorithm and the synergistic algorithm. In synergistic learning, the activation functions of neurons in both the hidden layer and the output layer are adjusted by IP. In this simulation, structural parameters of the FNN are set to 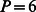 and 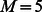. A Gaussian kernel is used to estimate entropy in all simulations with kernel size 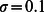. The initial values of all activation functions are set to 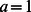 and 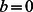. Figure 3 shows the learning curves of quadratic information potentials of the training error and Fig. 4 shows the learning curves of the training MSE. When calculating the MSE during the training procedure, the bias of the output is adjusted so as to cancel the mean error over the training set. For each learning curve, we display 300 epochs to compare the training speed and also display 1000 epochs to measure the final performance. As the learning curves of the information potential and the MSE show, the synergistic algorithm outperforms the MEE algorithm with regard to the convergence speed. After a long run, i.e., 1000 epochs, the synergistic algorithm can still maintain good performance. As a classical performance criterion, the mean square errors of the training set and the testing set after the 1000-epoch training process are summarized in Table 1 for “MG” and Table 2 for “SS”. In the last row of each table, the improvement percentage of the performance is the difference between the MSE of the MEE algorithm and the MSE of the synergistic algorithm, divided by the MSE of the MEE algorithm. According to the training and testing results of the MSE, the synergistic learning algorithm performs better than the MEE algorithm considered in isolation. The quadratic information potentials are also summarized in these tables. The improvement of the quadratic information potential is not as significant as that of the MSE.

**Table 1:** Performance comparison for the FNN using “MG”.

Data set	Training set		Testing set	
Criterion	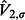	MSE	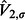	MSE
No IP	2.2632	0.0056670	2.2967	0.0051939
With IP	2.3950	0.0048786	2.4212	0.0044354
Improvement (MSE)	13.91%		14.60%	

**Table 2:** Performance comparison for the FNN using “SS”.

Data set	Training set		Testing set	
Criterion	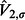	MSE	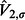	MSE
No IP	2.1518	0.0074291	2.6066	0.0019096
With IP	2.1905	0.0067886	2.6703	0.0012658
Improvement (MSE)	8.62%		33.71%	

**Figure 5:**
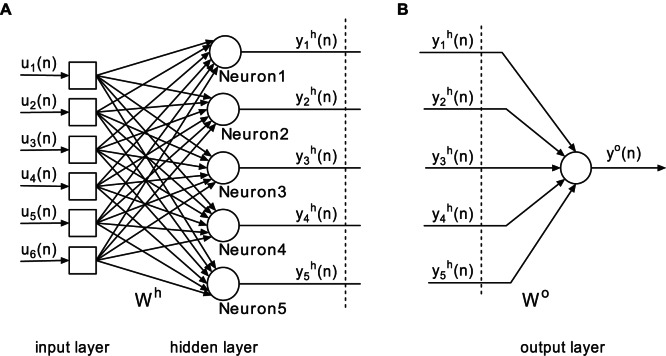
Decomposition of the FNN. (A) The input layer and the hidden layer of the FNN. (B) The output layer of the FNN.

In order to analyze the synergies between IP and synaptic plasticity in detail, input, output, and error distributions of neurons for the training set “MG” are presented. All these distributions are obtained by kernel density estimation in a single run, and results of two independent runs are similar. In order to explain the results clearly, we decompose the FNN into two parts, which are shown in Fig. 5.

**Figure 6:**
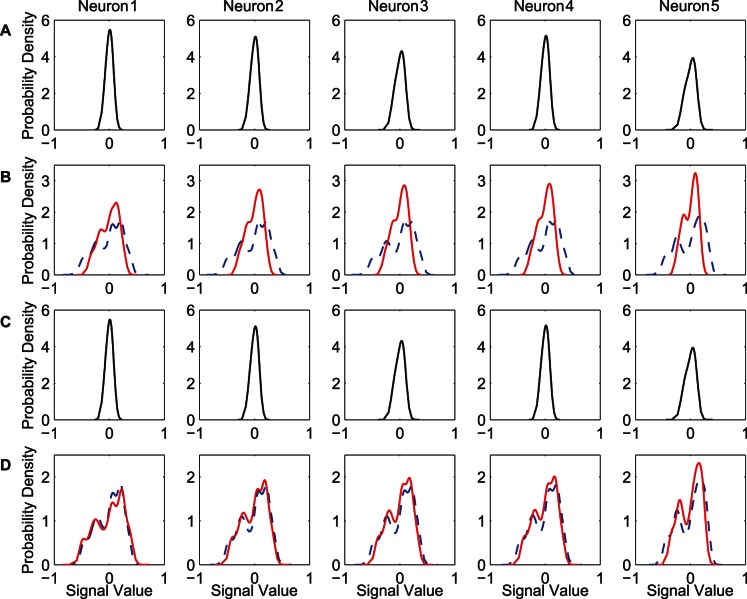
Input and output distributions for neurons in the hidden layer of the FNN. Input and output distributions for the five hidden neurons with the training data set “MG” are displayed. (A) Initial input distributions for the five hidden neurons. (B) Input distributions after 1000-epoch training for the two algorithms. (C) Initial output distributions for the five hidden neurons. (D) Output distributions after 1000-epoch training for the two algorithms. In (B) and (D), the dash lines denote the distributions obtained by the MEE algorithm, and the solid lines denote the distributions obtained by the synergistic algorithm.

Figure 6 shows the input and output distributions of the neurons in the hidden layer. We can refer to Figure 5(A) while analyzing the results shown in Fig. 6. Figure 6(A) shows the initial input distributions for five hidden neurons. As the elements of the initial weight matrices are randomly selected small values, the initial input of each hidden neuron is concentrated on a relatively small range. Figure 6(B) shows the input distributions after 1000 epochs, which are expanded into a relatively wide range in contrast to the initial input distributions. During the training process, the network input vectors 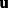 of each epoch for the two algorithms are totally identical, therefore the change of the input distributions of five hidden neurons is due to the updating of the synaptic weights 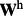. After training, the input distributions for the synergistic algorithm are more similar to the initial input distributions than those for the MEE algorithm, in other words, the change of the input distributions for the synergistic algorithm is relatively small. We infer that it is relatively easy for the weight update rule to form such input distributions from the initial input distributions, thereby accelerating the training process. Figure 6(C) shows the initial output distributions of the neurons in the hidden layer. The initial output is also concentrated on a small range. Figure 6(D) shows the output distributions after 1000 epochs. After training, the output distributions of the two different algorithms are similar for each hidden neuron.

**Figure 7:**
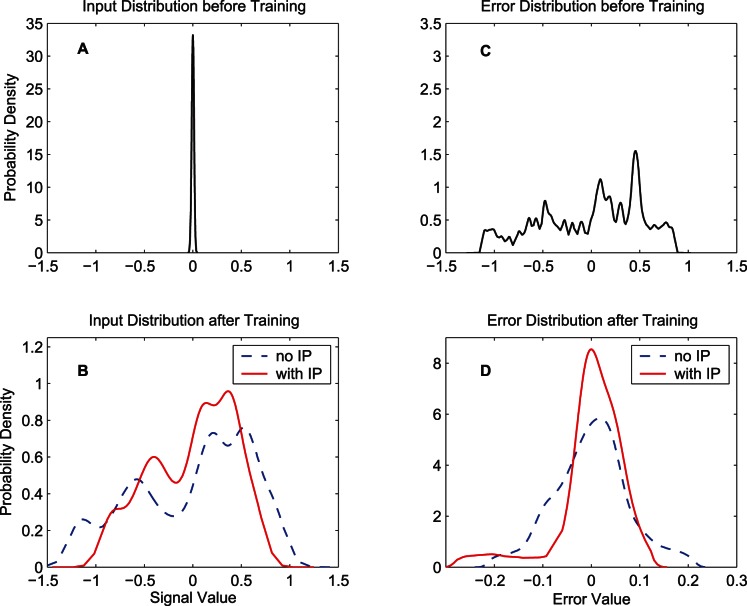
Input distributions for the output neuron and error distributions of the FNN. Input distributions for the single output neuron and error distributions with the training data set “MG” are presented. (A) Initial input distribution. (B) Input distributions after 1000-epoch training for the two algorithms. (C) Initial error distribution. (D) Error distributions after 1000-epoch training for the two algorithms. In (B) and (D), the dash lines denote the distributions obtained by the MEE algorithm, and the solid lines denote the distributions obtained by the synergistic algorithm.

Figure 7 shows the input and training error distributions of the output neuron. Since the output of this neuron is closely related to the error, so we do not present the output distribution here. We can refer to Fig. 5(B) while analyzing the results shown in Fig. 7. Figure 7 (A) and (B) show the input distributions of the output neuron (distributions of 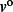) before and after training, respectively. Before training, the input distribution is concentrated around zero. After 1000 epochs, the input distribution obtained by the synergistic learning algorithm is more concentrated than the distribution obtained by the MEE algorithm. Note that the input of the output neuron 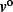 is the weighted sum of the output of hidden neurons, 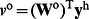. As we see from Fig. 6(D), the output distributions of each hidden neuron for the two algorithms are similar, which means that the statistical properties of the output of hidden neurons 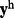 are similar in the two algorithms. Thus, the main reason for the difference in the distributions of 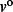 after training is the updating of the synaptic weights 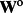. In the synergistic learning case, the distribution of 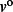 after training is relatively similar to the initial input distribution. This implies the weight updating process of the synergistic algorithm makes relatively small change of the synaptic weights 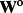, thus it is more easily achieved. Figure 7(C) and (D) show the error distributions of the two algorithms. After 1000 epochs, the FNN trained by the synergistic learning algorithm produces errors that are more concentrated around zero, which means there are higher number of small errors and fewer number of large errors, indicating better performance in terms of error. Although the increase of the quadratic information potential with IP is not substantial as shown in Table 1 and Table 2, learning with or without IP yields qualitatively different error distributions.

Thus, the analysis on the results in Fig. 6 and Fig. 7 provides an explanation for the fast convergence and good final performance of the synergistic learning shown in Fig. 3 and Fig. 4.

**Figure 8:**
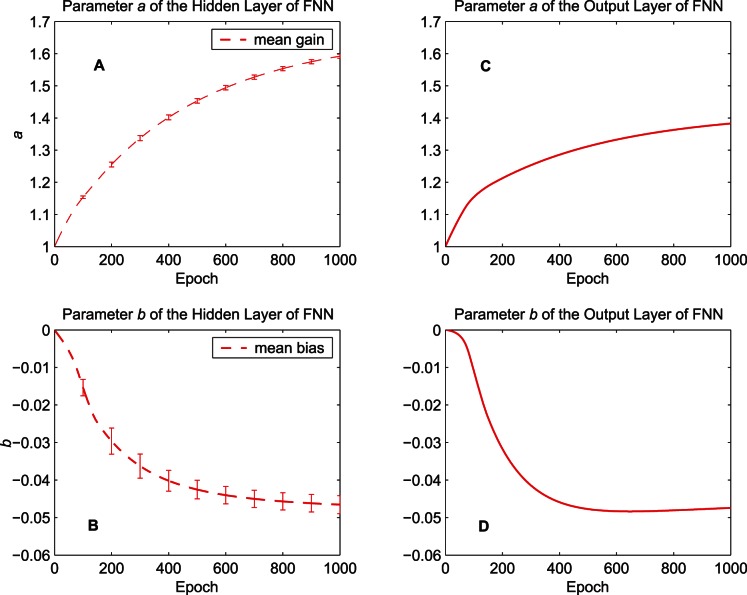
Evolution of the parameters of the activation functions in the FNN. The training data set “MG” is used. (A) Mean of the gain parameter 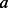 of the five hidden neurons. (B) Mean of the bias parameter 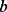 of the five hidden neurons. (C) The gain parameter 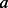 of the output neuron. (D) The bias parameter 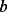 of the output neuron.

Figure 8 shows how the parameters of the activation functions in the FNN evolve over 1000 epochs. Figure 8 (A)(B) display the evolution of 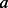 and 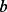 averaged over the five hidden neurons, respectively. The changes of the parameter pairs for the five hidden neurons are very similar, thus we show the averaged results and the corresponding error bars (standard deviations across the five hidden neurons). Figure 8 (C)(D) display the evolution of the parameters of the output neuron. In the FNN trained by the algorithm with IP, the values of 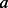 get large constantly to steepen the activation functions while the values of 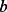 decrease.

**Figure 9:**
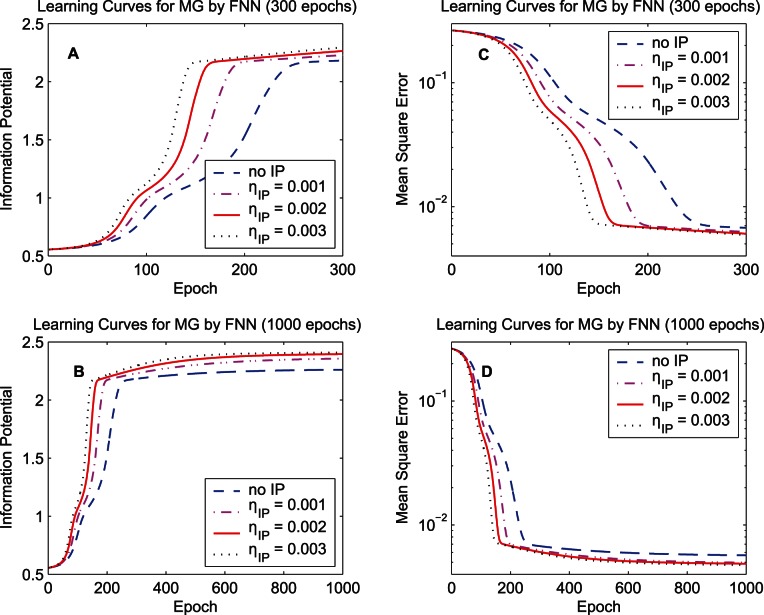
Learning curves of the FNN with different IP learning rates. The training data set “MG” is used. The initial IP learning rates 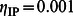, 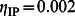, 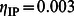, and 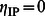 (no IP) are used for comparison. Learning curves of the quadratic information potential: (A) 300 epochs. (B) 1000 epochs. Learning curves of the mean square error: (C) 300 epochs. (D) 1000 epochs.

The second simulation concerns the IP learning rate. Figure 9 compares the learning curves of the synergistic algorithm with various initial IP learning rates 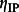 for the data set “MG”. Four initial IP learning rates 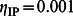, 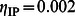, 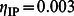, and 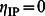 (no IP) are used for comparison. The results in Fig. 9 indicate that with a relatively large IP learning rate the information potential increases and the MSE decreases faster, but the highest information potentials and the smallest mean-square-errors during training procedures with the three non-zero IP learning rates are similar. In terms of the convergence speed, a relatively large IP learning rate is preferable, however, if 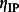 is set to be a much larger value (larger than 0.003), some ripples appear when the learning curve tends to converge, which is similar to that of the online reservoir adaptation by intrinsic plasticity in [21].

**Figure 10:**
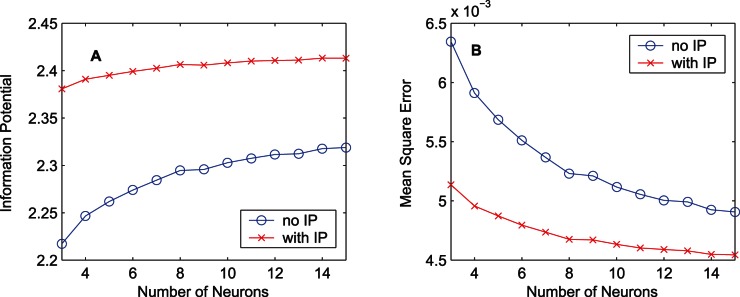
Relation between the training result and the number of hidden neurons of the FNN. Training results after 1000-epoch training for the case of the training data set “MG” are presented. The circle markers denote the results obtained by the MEE algorithm, and the cross markers denote the results obtained by the synergistic algorithm. (A) Results of the quadratic information potential. (B) Results of the mean square error.

Figure 10 displays the performance of FNNs with different numbers of hidden neurons (from 3 to 15) using the training data set “MG”. The values of the estimated quadratic information potential and the mean square error after 1000 epochs are shown. With the assistance of IP, the performance of the FNN containing various numbers of hidden neurons is always better. In terms of the quadratic information potential, the result obtained by a FNN containing 3 hidden neurons with IP is better than that obtained by a FNN containing 15 hidden neurons without IP. As for the MSE, the result obtained by a FNN containing 3 hidden neurons with IP is comparable to that obtained by a FNN containing 10 hidden neurons without IP. The performance improvement caused by adding IP is more significant than that caused by increasing the number of hidden neurons. In addition, increasing the number of hidden neurons brings a heavier computational burden. In the FNN, adding one hidden neuron brings extra 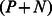 connection weights; for example, if we increase the number of hidden neurons from 5 to 10 in the above simulations, we have to update extra 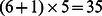 weights. However, we only need to update 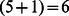 parameter pairs of 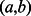 with simple calculations if we add IP to these five hidden neurons and the one output neuron. According to these results, the FNN trained by the synergistic learning algorithm can work well with fewer neurons and thus reduce the computational cost.

### Results of the RNN

As in the case of the FNN, we discuss how the recurrent neural network handles the problem of single-step prediction using the same data sets. The input of the network consists of 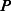 time-delay signals and 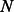 feedback signals,
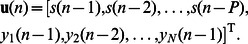
The prediction made by the recurrent neural network at time 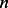 is the output of the first neuron 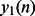, and the desired output is also 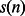.

The values of elements in the initial weight matrix are also randomly selected as small values uniformly distributed in [0, 0.05]. Results in this section are also averaged over 10 independent runs. The MEE learning rate is set to 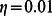. The initial IP learning rate is 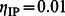 and it decreases exponentially, 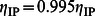.

**Figure 11:**
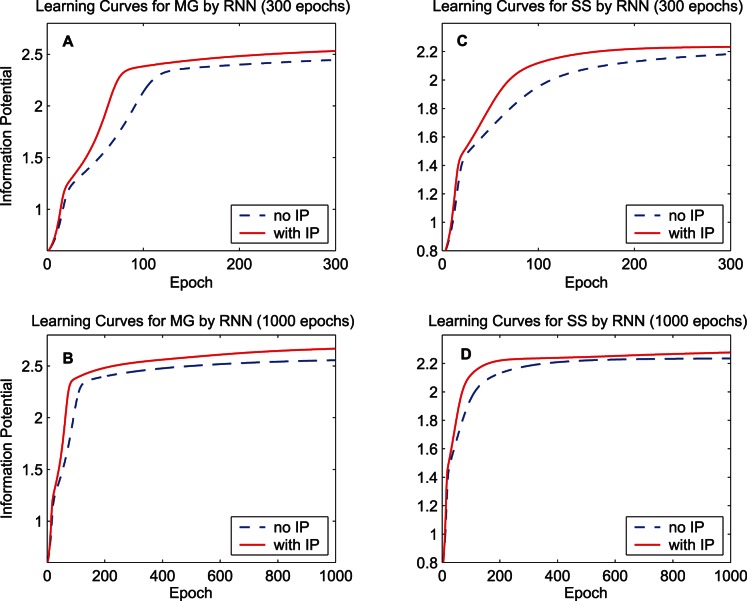
Learning curves of the quadratic information potential by the RNN. The dashed lines denote the learning curves of the MEE algorithm, and the solid lines denote the learning curves of the synergistic algorithm. (A) 300-epoch learning curves for the training data set “MG”. (B) 1000-epoch learning curves of “MG”. (C) 300-epoch learning curves for the training data set “SS”. (D) 1000-epoch learning curves of “SS”.

**Figure 12:**
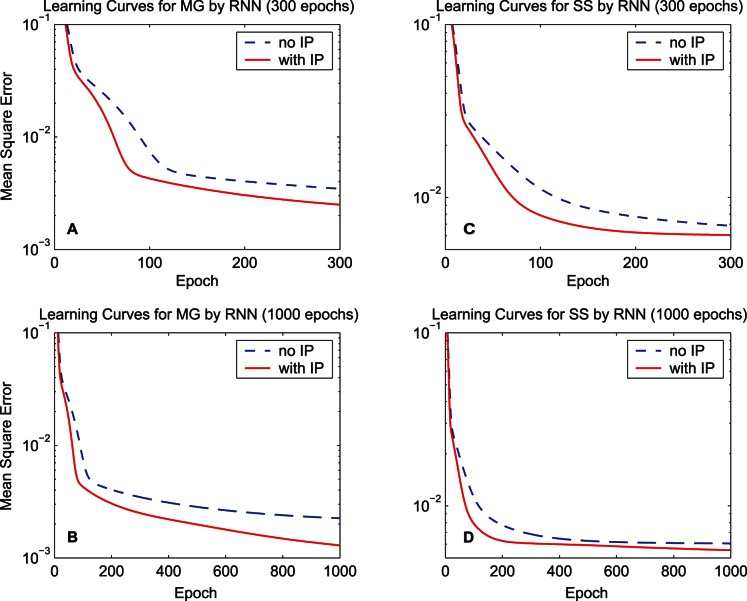
Learning curves of the mean square error by the RNN. The dashed lines denote the learning curves of the MEE algorithm, and the solid lines denote the learning curves of the synergistic algorithm. (A) 300-epoch learning curves for the training data set “MG”. (B) 1000-epoch learning curves of “MG”. (C) 300-epoch learning curves for the training data set “SS”. (D) 1000-epoch learning curves of “SS”.

The first simulation compares the learning curves of the synergistic algorithm and the MEE algorithm. Structural parameters of the RNN are set to 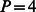 and 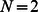. A Gaussian kernel with kernel size 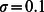 is used to estimate entropy. The initial values of all activation functions are set to 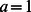 and 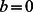. Figure 11 shows the learning curves concerning the quadratic information potentials of the training error, and Fig. 12 shows the learning curves of the training MSE. After the training procedure, the mean square errors and the quadratic information potentials of the training set and the testing set are summarized in Table 3 for “MG” and Table 4 for “SS”. These results manifest that the synergistic algorithm also outperforms the MEE algorithm for the RNN.

**Table 3:** Performance comparison for the RNN using “MG”.

Data set	Training set		Testing set	
Criterion	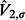	MSE	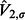	MSE
No IP	2.5666	0.0021446	2.5876	0.0019388
With IP	2.6653	0.0013974	2.6994	0.0009594
Improvement (MSE)	34.84%		50.51%	

**Table 4:** Performance comparison for the RNN using “SS”.

Data set	Training set		Testing set	
Criterion	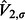	MSE	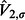	MSE
No IP	2.2362	0.0060375	2.6811	0.0011350
With IP	2.2765	0.0055386	2.7057	0.0009260
Improvement (MSE)	8.26%		18.41%	

**Figure 13:**
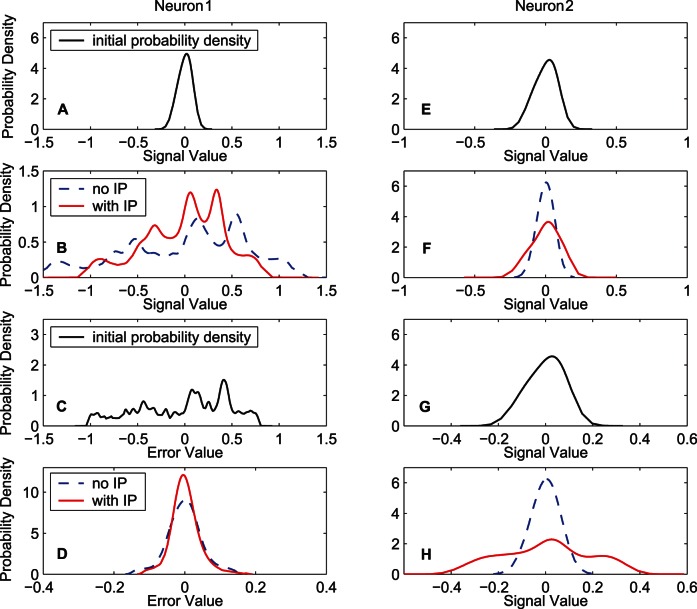
Input, output and error distributions for neurons of the RNN. The training data set “MG” is used. Neuron 1 (output neuron): (A) Initial input distribution. (B) Input distributions after 1000-epoch training for the two algorithms. (C) Initial error distribution. (D) Error distributions after 1000-epoch training for the two algorithms. Neuron 2: (E) Initial input distribution. (F) Input distributions after 1000-epoch training for the two algorithms. (G) Initial output distribution. (H) Output distributions after 1000-epoch training for the two algorithms. In (B), (D), (F), and (H), the dash lines denote the distributions obtained by the MEE algorithm, and the solid lines denote the distributions obtained by the synergistic algorithm.

Figure 13 shows the input and output distributions of the neurons in the RNN. Before training, the input distribution of the first neuron (the output neuron of the RNN, denoted by “Neuron 1″ in the figure) is concentrated on a small range, as shown in Fig. 13(A). After training, in contrast to the situation without IP, the change of the input distribution from the initial state is relatively small in the situation with IP, as shown in Fig. 13(B). The training error distributions before and after training are shown in Fig. 13(C) and (D), respectively. The situation of the first neuron in the RNN is similar to that of the output neuron in the FNN, but the difference between the distributions with and without IP for the second neuron (denoted by “Neuron 2″ in the figure) seems interesting. Without IP, the input and output distributions of the second neuron after learning are restricted in a relatively small range. However, with IP, the input distribution after learning is expanded to a wider range; correspondingly, the output of the second neuron is also expanded from the initial distribution. Since the output signal returns to constitute the input of the RNN, if the output of the second neuron is restricted in a very small range, the input signal, 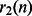, is ineffective. With a wide data range, 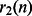 can provide more information (larger entropy) for the input of the RNN.

**Figure 14:**
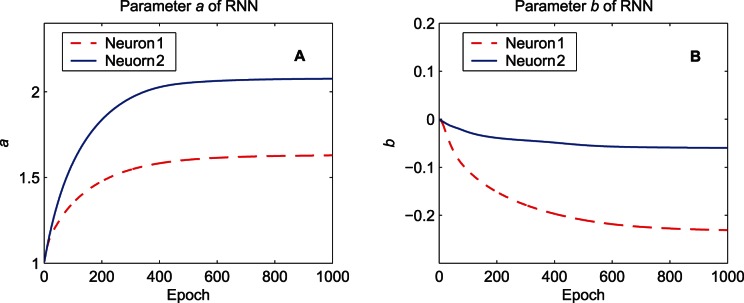
Evolution of the parameters of the activation functions in the RNN. The training data set “MG” is used. (A) The gain parameter 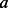. (B) The bias parameter 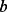.

Figure 14 shows the evolution of the parameters of the activation functions in the RNN. The values of 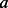 get large constantly to steepen the activation functions while the values of 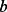 of the two neurons are adjusted to match the position of the input distribution.

**Figure 15:**
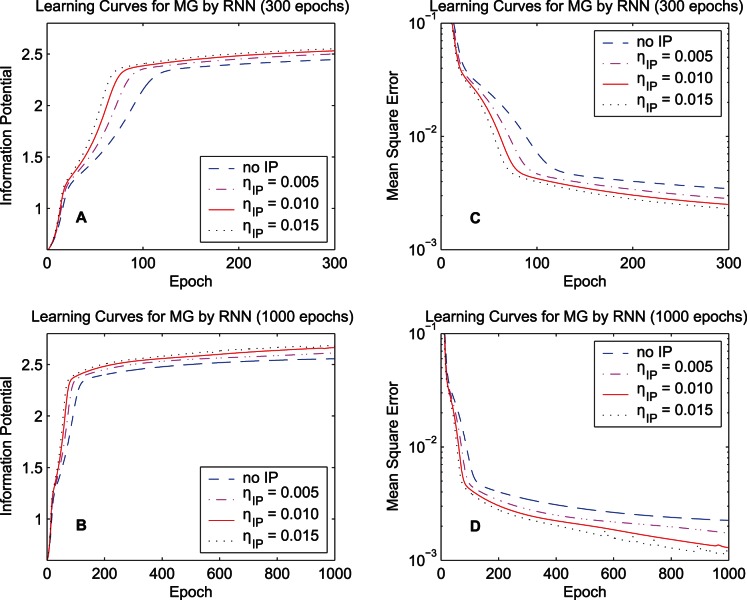
Learning curves by the RNN with different IP learning rates. The training data set “MG” is used. The initial IP learning rates 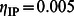, 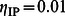, 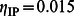, and 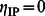 (no IP) are used for comparison. Learning curves of the quadratic information potential: (A) 300 epochs. (B) 1000 epochs. Learning curves of the mean square error: (C) 300 epochs. (D) 1000 epochs.

The second simulation concerns the initial IP learning rate. Figure 15 compares the learning curves of the synergistic algorithm with various initial IP learning rate 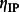 for the training data set “MG”. The initial IP learning rates 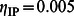, 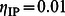, 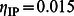, and 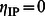 (no IP) are used. In terms of both the convergence speed and the final result, a relatively large IP learning rate is better; however, oscillation behavior appears when 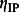 gets much larger. This phenomenon of the intrinsic plasticity rule may be ubiquitous in different kinds of neural networks.

**Figure 16:**
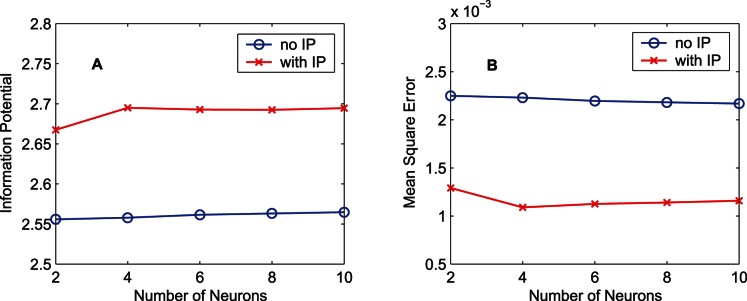
Relation between the training result and the number of neurons of the RNN. Training results after 1000-epoch training for the case of the training data set “MG” are presented. The circle markers denote the results obtained by the MEE algorithm, and the cross markers denote the results obtained by the synergistic algorithm. (A) Results of the quadratic information potential. (B) Results of the mean square error.

Figure 16 displays the performance of the RNNs with different numbers of neurons using the training data set “MG”. Without IP, the performance improvement is trivial with the increasing of the number of neurons. The MEE learning algorithm for the RNN seems insensitive to the number of neurons. In this situation, using two neurons seems effective enough since adding neurons increases the computational cost but neither raises the quadratic information potential nor lowers the MSE substantially. For the RNNs, the performance improvement caused by adding IP is far more significant than that caused by increasing the number of hidden neurons.

In some of the above simulations, we do not show the results for the data set “SS”, since they are similar to those for the data set “MG”. All activation functions used in the above-mentioned neural networks are tanh functions, thus the intrinsic plasticity rule for the tanh function is applied to the synergistic learning algorithm. In the case of using logistic functions, similar results can be obtained.

## Discussion

Combining the MEE algorithm as the synaptic plasticity rule and the information-maximization algorithm as the intrinsic plasticity rule, we proposed a synergistic information-theoretic learning algorithm for training artificial neural networks. Whereas the information-maximization algorithm can increase the mutual information of a single neuron, it can not optimize the cost function such as EEC and MSE. Nevertheless, simulations have shown that this information-maximization-based IP rule benefits the artificial neural networks in both the convergence speed and the final learning result. As the IP rule adjusts the activation function of a single neuron to match its input distribution so that all output levels tend to appear equivalently, the input can be encoded much more efficiently and the discriminative ability of the neuron is enhanced. We believe that the discriminative ability of a neuron plays a nontrivial role in the performance of artificial neural networks. In terms of the FNN, the synergistic learning algorithm with IP only in the hidden layer or only in the output neuron still outperforms the MEE algorithm without IP, but is inferior to the learning algorithm with IP in both layers (we do not present these results in the paper).

Compared with the original algorithm, the synergistic learning algorithm can be performed with a relatively small increase in computational cost due to the local nature of the IP mechanism and the simplicity of the information-maximization algorithm. In addition, we have used the efficient batch version of the information-maximization algorithm. In applications, a long training process is unnecessary since the improvement is minor at the end part of the training. For example, with a 300-epoch training, the IP rule is quite effective to improve the performance. In a long run, the synergistic learning maintains good performance.

Advanced search methods for nonlinear optimization such as conjugate gradient algorithms and the Levenberg-Marquardt algorithm can be used to further speed up the learning process. In order to focus on the synergies between IP and synaptic plasticity and preclude influences of other advanced search methods on learning, we used the simple gradient descent (GD) method.

In biology, Bell and Sejnowski's information-maximization algorithm can match the statistics of naturally occurring visual contrasts to the response amplitudes of the blowfly's large monopolar cell (LMC). The contrast-response function of the LMCs in the blowfly's compound eye approximates to the cumulative probability distribution of contrast levels in natural scenes, thus the inputs are encoded so that all response levels are used with equal frequency, resulting in a uniform output distribution [28]. We may regard this experimental result as the biological justification of the proposed synergistic learning rule.

As related work, several studies have combined synaptic learning algorithms with Triesch's IP rule [10], [11], [29] or other revised versions for training artificial neural networks. In [30], an unsupervised scheme including the IP rule for pretraining extreme learning machines is introduced. In [21], [31], an online adaptation rule with IP for the reservoir networks is presented. To the best of our knowledge, all these previous studies on the effects of IP on neural network learning have used the MSE criterion rather than the EEC criterion [21], [30], [31]. Besides, the energy consumption of a biological neuron is considered as an important constraint for the IP rules used in these previous studies. In this study, we neglect this energy constraint and regard Bell and Sejnowski's information-maximization algorithm for a single neuron's activation function as the intrinsic plasticity rule. In a recent study related to ours, Lazar et al. presented a self-organizing recurrent network (SORN) combining intrinsic plasticity and synaptic plasticity that learns spatio-temporal patterns in its input while maintaining its dynamics in a healthy regime suitable for learning, in which the IP rule regulates a neuron's firing threshold to maintain a low average activity level and the synaptic rule is a simple model of STDP [29]. This work implies that as we try to understand neural plasticity and how it shapes the brain's representation and processing, it is insufficient to study individual mechanisms in isolation and studying their interactions is necessary [29]. In this study, we have shown how the information-maximization IP rule improves the performance of FNNs and RNNs trained with the EEC criterion and we draw the conclusion that the interactions of different plasticity mechanisms can benefit artificial neural networks in supervised learning applications. Here we have focused on providing an upgraded information-theoretic learning method for applications and we have not specifically attempted to emphasize on the biological relevance.
